# Point-of-care Ultrasound Used in the Diagnosis of Reverse Takotsubo Cardiomyopathy

**DOI:** 10.5811/cpcem.20811

**Published:** 2024-11-04

**Authors:** Pooja Dave, Annemarie Daecher, Claire Abramoff

**Affiliations:** * Albert Einstein Medical Center, Department of Emergency Medicine, Philadelphia, Pennsylvania; † Georgetown University Medical Center, Division of Pulmonary and Critical Care Medicine, Washington, District of Columbia

**Keywords:** *basal hypokinesis*, *catecholamine surge*, reverse takotsubo cardiomyopathy

## Abstract

**Case Presentation:**

We present a case of a 50-year-old patient who presented to the emergency department with palpitations, nausea, vomiting, and chest discomfort. She was found to have a reduced ejection fraction and basal wall hypokinesis on point-of-care ultrasound concerning for reverse takotsubo cardiomyopathy.

**Discussion:**

Reverse takotsubo cardiomyopathy is a rare variant of takotsubo cardiomyopathy and involves basal ballooning instead of apical ballooning. Ultrasound findings concerning for reverse takotsubo cardiomyopathy are basal wall hypokinesis or akinesis.

## CASE PRESENTATION

A 50-year-old female with a past medical history of COVID-19-induced myocarditis, asthma, and a now-resolved left ventricular thrombus presented to the emergency department (ED) for two days of intermittent heart palpitations, chest discomfort, nausea, and vomiting. Her initial vital signs in the ED were a temperature of 36.5° Celsius, heart rate of 115 beats per minute, blood pressure of 145/64 millimeters of mercury, and oxygen saturation of 95% on room air. Pertinent physical exam findings included a regular heart rate and rhythm, with no murmurs. Her initial electrocardiogram (ECG) was significant for new ST-segment depressions laterally, which resolved on a repeat ECG four hours later. Troponin was initially 1.13 nanograms per milliliter (ng/mL) (normal range 0.00–0.03 ng/mL) and trended to 0.66 ng/mL four hours later and 0.22 ng/mL two days later. A point-of-care ultrasound was performed, which revealed decreased wall motion at the heart base with a decreased ejection fraction (Supplemental Videos 1–3).

Computed tomography of the chest, abdomen, and pelvis with contrast did not show signs of aortic pathology. Given concern for non-ST-segment elevation myocardial infarction and reverse takotsubo cardiomyopathy, the patient was admitted to telemetry for a formal echocardiogram and monitoring for three days. She received aspirin 81 milligrams (mg), ondansetron, and morphine in the ED. Her formal echocardiogram showed a mildly enlarged left ventricle and moderately reduced left ventricular systolic function with an ejection fraction 35–40% (normal ejection fraction > 55%), as well as grade 1 diastolic dysfunction. The basal to mid anteroseptal, basal to mid inferoseptal, basal anterior, and basal to mid inferior and basal inferolateral walls were found to be akinetic, concerning for reverse takotsubo cardiomyopathy. The patient was ultimately discharged from the hospital with metoprolol extended-release 25 mg daily and losartan 50 mg daily. At the time of discharge, she had overall improvement of her chest pain and nausea but stated that it intermittently came back. Of note, she did endorse being anxious and stressed due to a family member requiring frequent hospitalizations. At a cardiology appointment one month after her presentation, her symptoms had resolved, and a cardiac magnetic resonance imaging was recommended.

## DISCUSSION

Takotsubo cardiomyopathy is thought to be a form of left ventricular dysfunction that is often triggered by emotional or physical stress. While the exact pathophysiology of takotsubo cardiomyopathy is unknown, it is secondary to a catecholamine surge.^2^ Multiple variants of takotsubo cardiomyopathy exist including reverse takotsubo cardiomyopathy, which is characterized by basal ballooning, instead of the more typical apical ballooning. Reverse takotsubo cardiomyopathy is relatively rare and is thought to make up 1–23% of takotsubo cardiomyopathy cases.^1^ It is more commonly seen in a younger population and had a higher prevalence of preceding emotional or physical stress compared to the other types of takotsubo cardiomyopathy.^3^,^4^ Patients with reverse takotsubo were also less likely to present with severe heart failure symptoms such as dyspnea and cardiogenic shock.^4^ Including reverse takotsubo in our differential and performing point-of-care ultrasound at the bedside can help guide further treatment and provide insight about the disease course and recovery.

**CPC-EM Capsule**

What do we already know about this clinical entity?

*Reverse takotsubo is a rare type of cardiomyopathy causing basal ballooning of the left ventricle. It is thought to be triggered by emotional or physical stress.*

What is the major impact of the image(s)?

*Reverse takotsubo classically presents with basal hypokinesis. The videos here depict basal hypokinesis in the subxiphoid and apical 4-chamber view.*

How might this improve emergency medicine practice?

*Visualizing examples of basal hypokinesis can improve a practitioner’s ability to recognize reverse takotsubo and thereby guide further treatment decisions.*

## Supplementary Information






